# Maghemite Nanoparticles Acts as Nanozymes, Improving Growth and Abiotic Stress Tolerance in *Brassica napus*

**DOI:** 10.1186/s11671-017-2404-2

**Published:** 2017-12-19

**Authors:** N.G. Martin Palmqvist, Gulaim A. Seisenbaeva, Peter Svedlindh, Vadim G. Kessler

**Affiliations:** 1 Department of Chemistry and Biotechnology, Swedish University Agricultural Sciences, Box 7015, SE-75007 Uppsala, Sweden; 2 Department of Engineering Sciences, Solid State Physics, Uppsala university, Box 534, SE-75121 Uppsala, Sweden

**Keywords:** Nanozyme, Maghemite nanoparticles, Drought stress, Nanofertilizer, Catalase activity, Iron oxide nanoparticles, Nano agriculture, Agrobio nanotechnology, Growth promotion, Reactive oxygen species scavenging

## Abstract

Yttrium doping-stabilized γ-Fe_2_O_3_ nanoparticles were studied for its potential to serve as a plant fertilizer and, through enzymatic activity, support drought stress management. Levels of both hydrogen peroxide and lipid peroxidation, after drought, were reduced when γ-Fe_2_O_3_ nanoparticles were delivered by irrigation in a nutrient solution to *Brassica napus* plants grown in soil. Hydrogen peroxide was reduced from 151 to 83 μM g^−1^ compared to control, and the malondialdehyde formation was reduced from 36 to 26 mM g^−1^. Growth rate of leaves was enhanced from 33 to 50% growth compared to fully fertilized plants and SPAD-measurements of chlorophyll increased from 47 to 52 suggesting improved agronomic properties by use of γ-Fe_2_O_3_ nanoparticles as fertilizer as compared to chelated iron.

**Electronic supplementary material:**

The online version of this article (10.1186/s11671-017-2404-2) contains supplementary material, which is available to authorized users.

## Background

Food security is of paramount importance and a pressing issue of our changing world. A changing climate and growing population are steering plant scientists and agricultural engineers to innovate improved tools to secure food production with less environmental impact. Nanotechnologies are one such novel tool that can be explored to solve this longstanding problem [1–3]. Nanotechnology has been predicted to become an important and integral part of the food production chain, serving, for example, a role in crop protection [4–6], fertilizers [7, 8], biosensors and precision farming [9], and food packaging and safety [10]. Nanoparticles are ubiquitous in nature, and plants have evolved exposed to various nanoparticles [11]. Iron oxide nanoparticles (IONs) constitute an important part of naturally occurring nanoparticles [12]. There is evidence that plants and soil microbes produce IONs [11, 13, 14]. While some researchers have been concerned with the toxicity to plants of engineered IONs [15, 16], others have focused on the possibility of using IONs as a fertilizer [17–22]. Magnetic nanoparticles of magnetite Fe_3_O_4_ and maghemite γ-Fe_2_O_3_ structure have been suggested to be effective nanozymes of both peroxidase mimetic ability (at low pH) and catalase mimetic ability (at neutral pH) [23–25]. It has been shown that, at certain concentrations, nano iron oxide increases plant growth compared to the addition of equivalent amounts of ferrous ions in chelated form [17]. We hypothesize that the enzymatic abilities of nano iron oxide can stimulate growth in plants above that of just iron fertilization. Further, we suggest that this should aid plants during common abiotic stresses such as drought, where catalase and peroxidase becomes important for scavenging of reactive oxygen species (ROS) being released. Here, we present investigations to test this hypothesis on γ-Fe_2_O_3_ and oil seed rape, grown in soil and controlled environment.

## Results

### Effect of Particles on Plant Traits

**Fig. 1:**
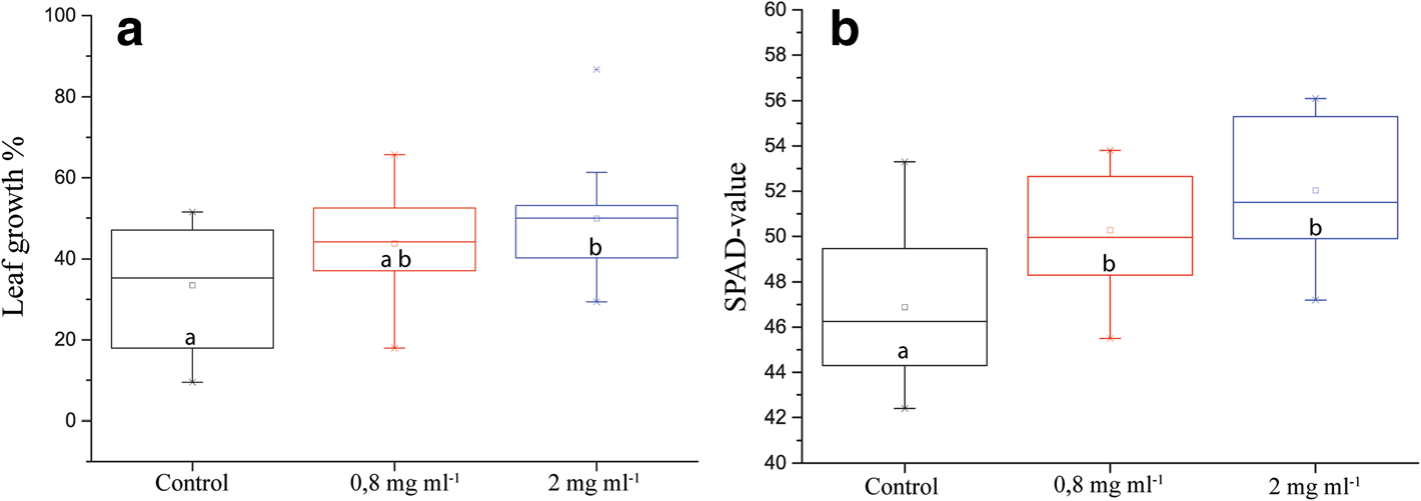
Different physiological parameters of plants grown in pots with soil irrigated with nutrients or nutrients containing IONs. **a** Individual leaf length increase from before until after 5 days of ION-treatment (*n* = 16, *p* value = 0.053). **b** Chlorophyll content in the leaves, as measured with SPAD measurement (*n* = 16, *p* value = 0.000). Different letters signify statistically significant difference

By adding IONs, we increased growth of oilseed rape compared to just adding an adequate amount of chelated iron. Leaf length showed a statistically significant increase compared to the control, suggesting an increase in either cell division or cell elongation (Fig. 1a). Before the plants were subjected to drought there was a statistically significant increase in chlorophyll content as measured by SPAD-meter, suggesting an increased fitness of these plants compared to control (Fig. 1b).

**Fig. 2:**
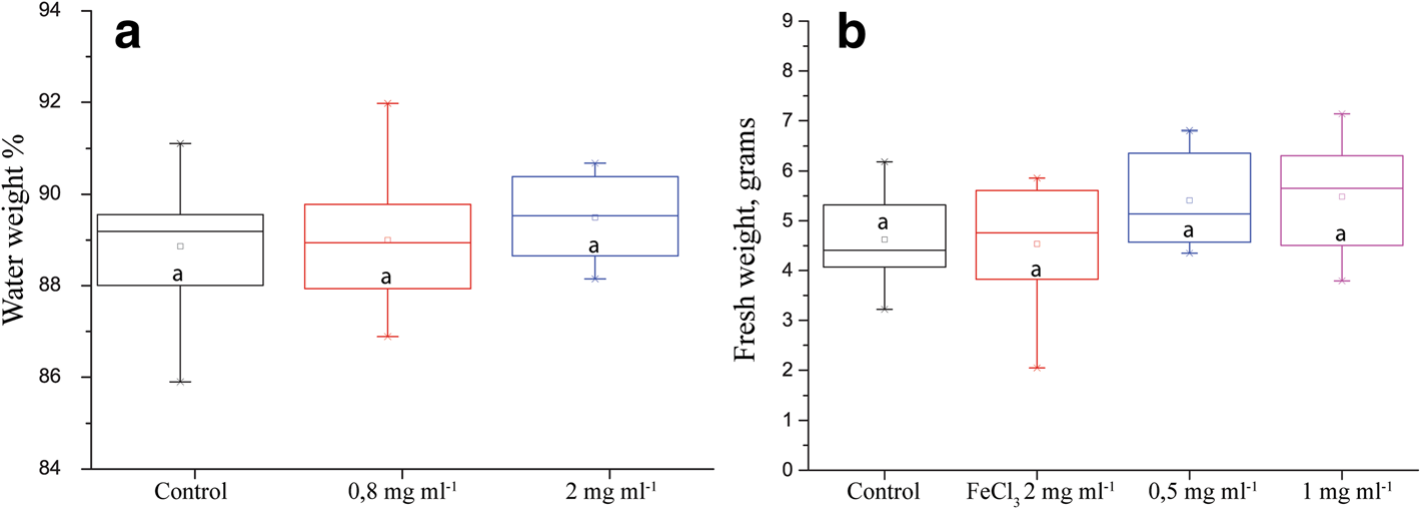
Plant parameters after drought stress. **a** Percent of plant weight that constitutes water. **b** Plant biomass after 5 days of drought (*n* = 8, *p* value = 0.127). Different letters signify statistically significant difference

**Fig. 3:**
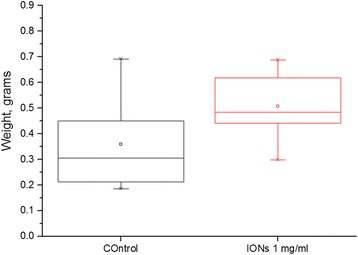
Fresh weight of plants, measured after 5 days of drought. Statistically significant difference with 15 biological replicates and *p* value 0.01

Water loss did not show statistically significant difference but there was a tendency towards greater water retention in treatments with IONs (Fig. 2a). Fresh weights, which also take into account the growth of the plants, always showed higher values for ION treatments (Fig. 2b) and were statistically significant in some cases. For example, one experiment with extended drought can be seen in Fig. 3.

**Fig. 4:**
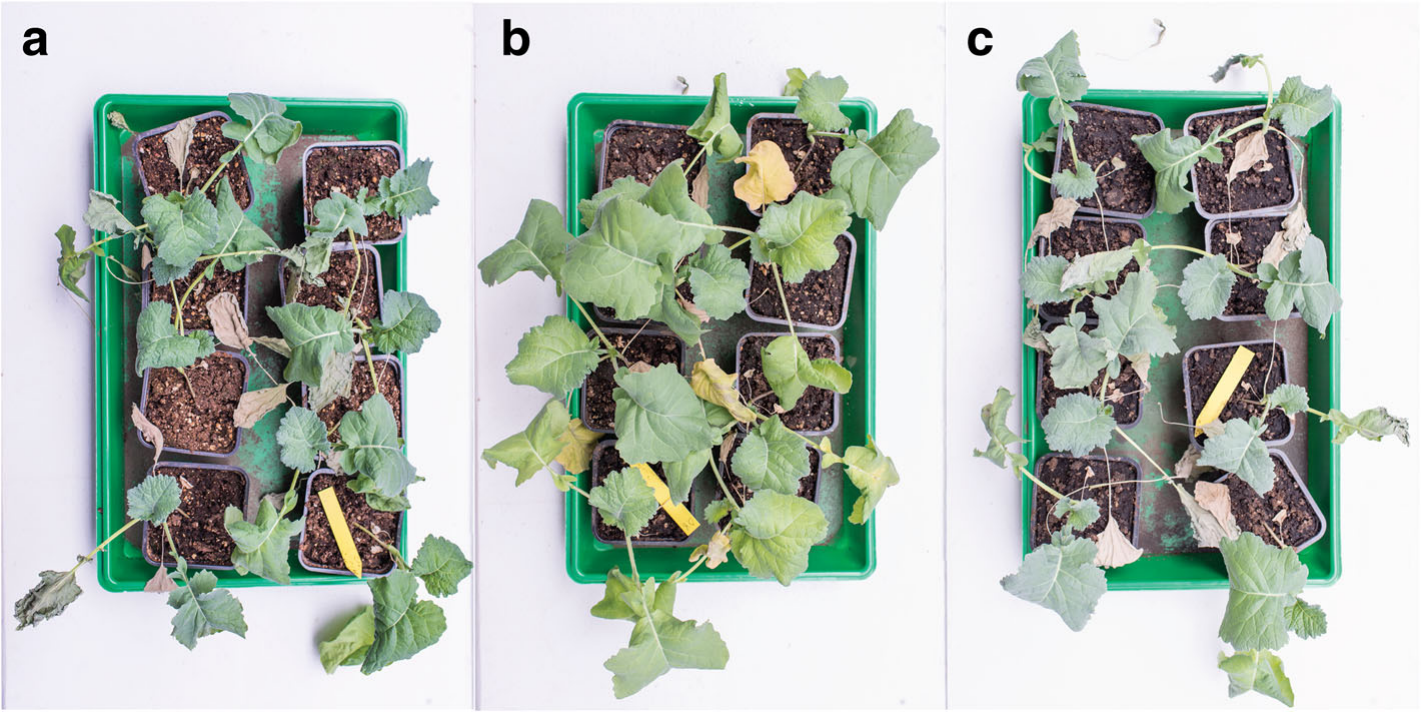
Photos of plants after rewatering after 5 days of drought stress. **a** Control plants irrigated with nutrient solution. **b** Plants irrigated with nutrient solution containing 0.8 mg/ml IONs. **c** Plants irrigated with nutrient solution containing 2 mg/ml IONs

Considering that control also has adequate amount of iron, big differences in fresh weight would be exceptional. It was observed that the plants treated with IONs coped better than control during drought and recovered better after rewatering (Fig 4).

### Effects of IONs on Leaf Hydrogen Peroxide Concentration

**Fig. 5:**
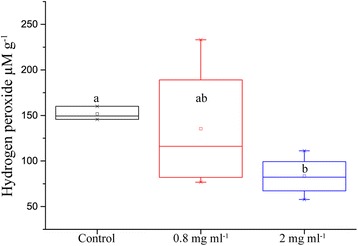
Amount of soluble hydrogen peroxide per gram of leaf tissue from oilseed rape treated with nutrient solution containing IONs and challenged with drought for 5 days (*n* = 16, *p* value = 0.004)

The amount of hydrogen peroxide in the leaf after drought was substantially reduced when IONs were added to the nutrient solution used for watering. Variation was high in the 0.8 mg ml^−1^ treatment; hence, difference towards the other treatments is not statistically significant. However, the difference between control and the highest concentration of 2 mg ml^−1^ is statistically significant with a *p* value of 0.004 and a mean 84% greater in the control treatment (Fig. 5).

### Effects of IONs on Lipid Peroxidation

**Fig. 6:**
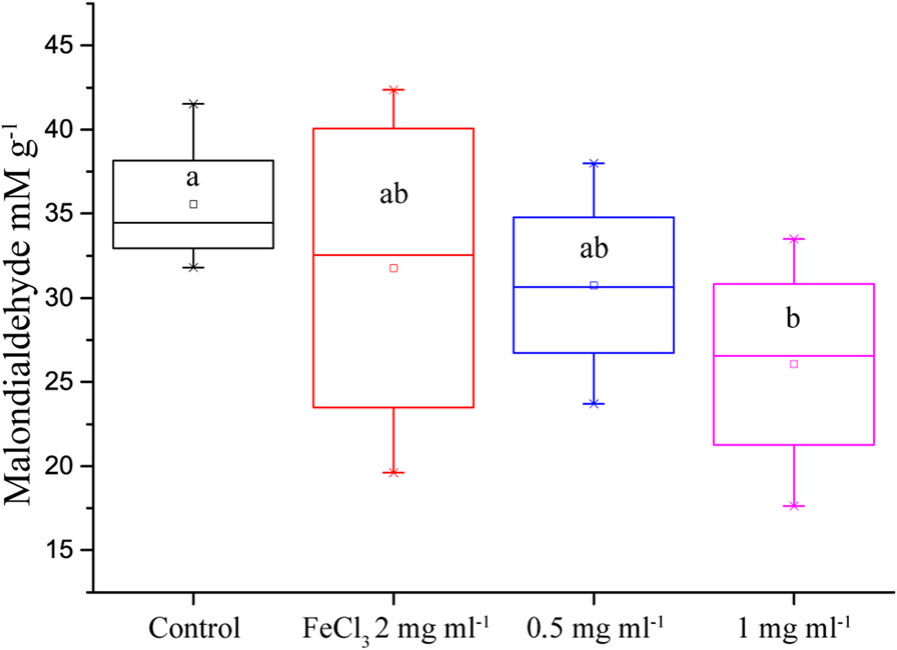
Concentration of the lipid peroxidation product MDA in the leaves of oilseed rape treated with nutrient solution containing IONs and drought for 5 days (*n* = 8, *p* value = 0.052)

Lipid peroxidation with MDA levels as proxy was reduced by addition of IONs, with 36% lower mean concentration of MDA in the leaves of plants with 200 mg of IONs added. We added a positive control with the same molar concentration of iron (III) ions; however, the variation was too big to make any conclusions. The mean of the lower ION concentration was also lower than control, showing a trend towards reduced lipid peroxidation in the leaves of oil seed rape (Fig. 6).

### Plant Particle Uptake

**Fig. 7:**
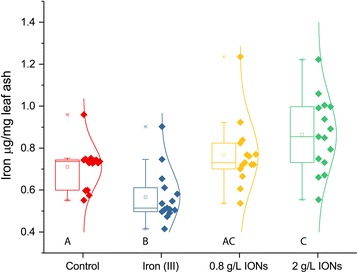
Iron concentration in brassica plant leaves after treatment with maghemite nanoparticles, as compared to control with same nutrient solution or the same nutrient solution with 1:1 M ratio of iron (III) ions. Different letters signify statistically significant difference (*n* = 15)

To investigate particle uptake into leaf tissue, we measured the iron content of the leaves with inductively coupled plasma atomic emission spectroscopy (ICP-AES). Indeed, a statistically significant increase of iron was observed in treatments with maghemite nanoparticles. Interestingly, the concentration of iron was reduced in leaves irrigated with superfluous iron (III) ions (Fig. 7).

**Fig. 8:**
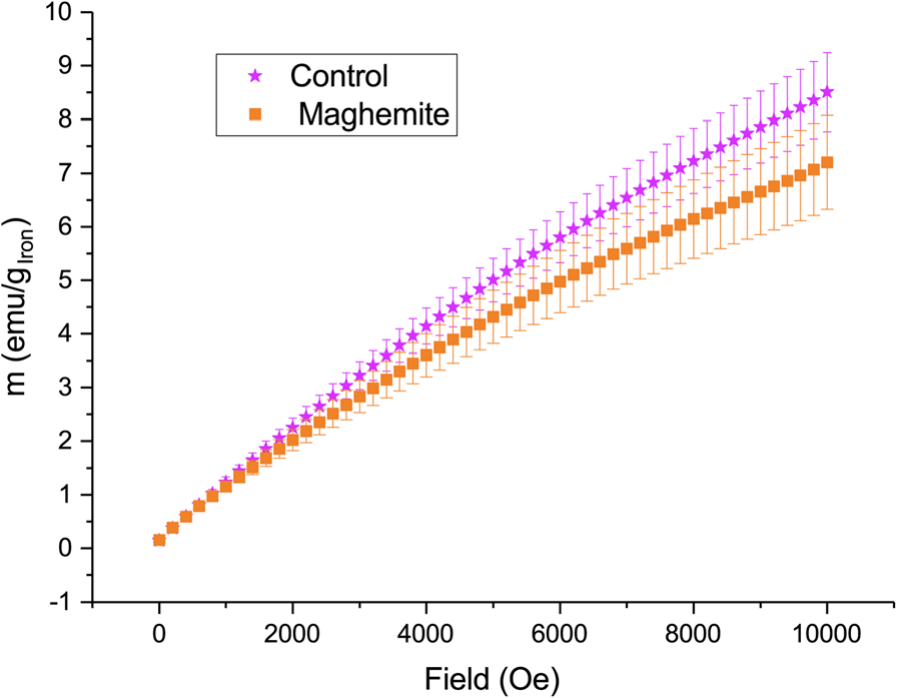
Low-temperature (2 K) magnetization of ashed leaves of plants treated with maghemite nanoparticles compared to control plants. Error bars show standard error of mean (*n* = 6)

**Fig. 9:**
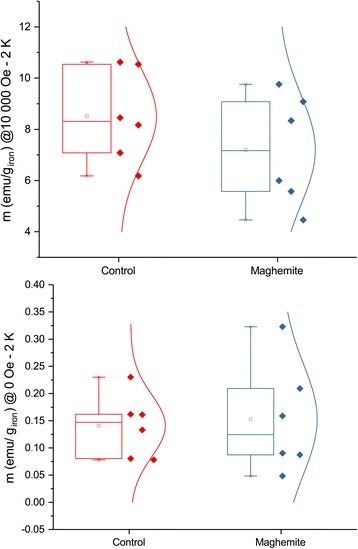
Low-temperature (2 K) magnetization of ashed leaves under different magnetic fields. The plot above at 10000 Oe have a *p* value of 0.8, and the plot below at zero field has a *p* value of 0.08 (*n* = 6)

To further corroborate the increased iron content as proof of particle uptake, we measured the low-temperature magnetization in the same leaves. A larger magnetization was observed in control under a strong magnetic field, but without magnetic field, the remanent magnetization was larger in leaves treated with maghemite nanoparticles (Figs. 8 and 9). Due to small sample size and great variation, the differences are not statistically significant but the trend clearly shows a presence of superparamagnetic IONs since the magnetization is higher in control under high magnetic fields but lower when there is no magnetic field. It is clouded by variation, but in certain samples, the presence of IONs was clearly visible (Additional file 1: Figure S2). On one hand, at low enough temperature and at high enough magnetic field, the magnetization for iron ions will be larger than that of ferrimagnetic IONs. On the other hand, at the same low temperature but at zero magnetic field, the remanent magnetization will be larger for IONs due to blocked nanoparticle magnetic moments.

### Material Characterizations

The IONs produced by the method of Cui et al. (2013) formed a gel, indicating successful production of nanoparticles on the order of ~ 1–10 s nm. The dried gel was ground into a powder. The low resolution SEM cannot show individual particles but the hierarchical structure of the powder is evident; the EDS of the sample did not detect any Y, only iron (Fig. 7).

When dispersed in water, the particles form aggregates, with a hydrodynamic size of up to 500 nm, however 84% of the aggregated particles are smaller than 300 nm and at least 11% are smaller than 50 nm. In absolute values, according to the Nanosight measurements, there are 4.28 × 10^6^ particles smaller than 20 nm ml^−1^, in the 50 times diluted dispersion needed for measurement (Fig. 8). Calculating back, that means that there are approximately 2 × 10^8^ particles smaller than 20 nm ml^−1^ in the treatments.

The images made by AFM show a similar pattern as the NTA combined with XRD vide infra, with particle sizes from a few nanometers to aggregates of several hundred nanometers (Fig. 9).

**Fig. 10:**
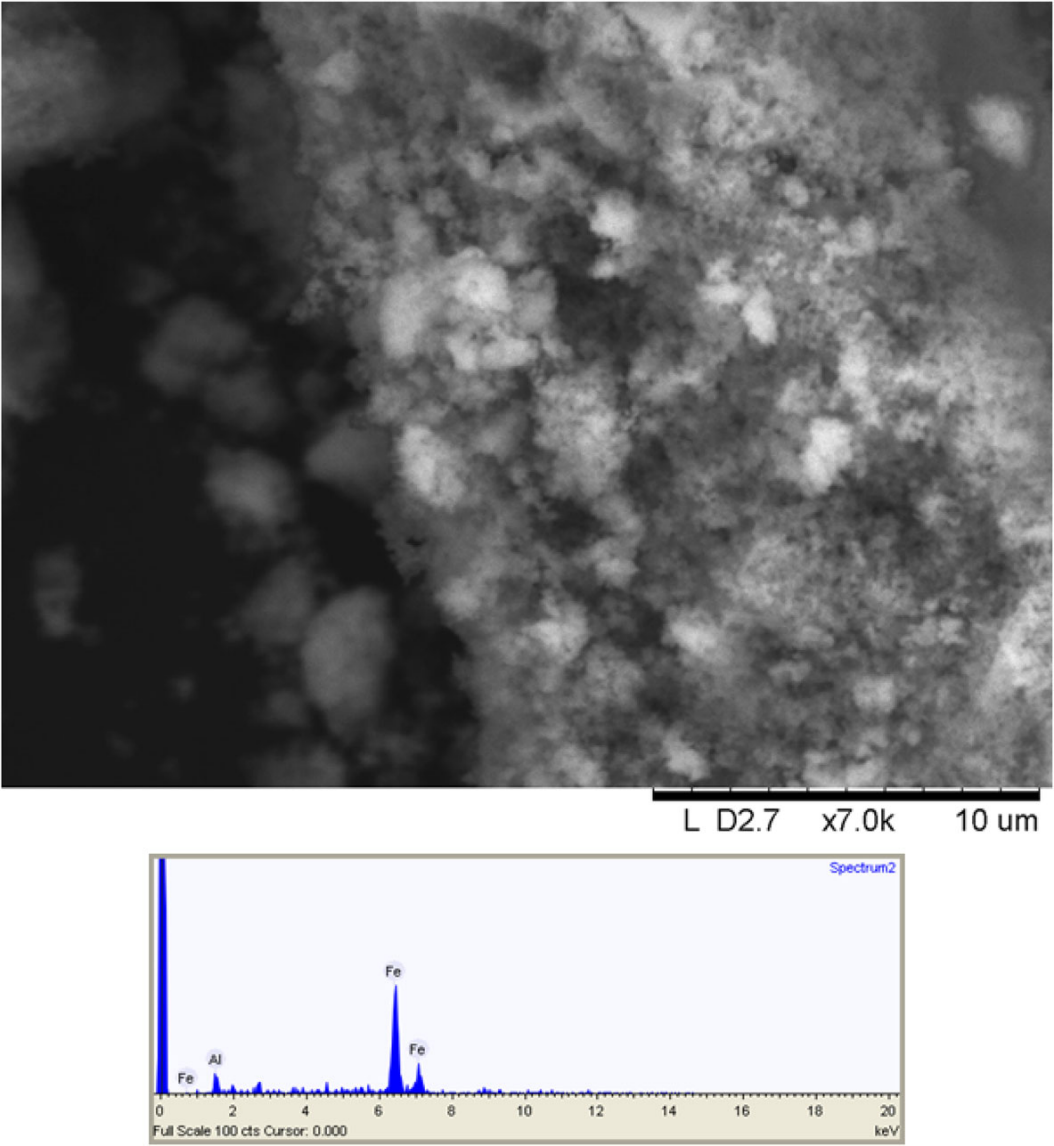
Scanning electron image of γ-Fe_2_O_3_ synthesized through yttrium directed sol-gel synthesis and an EDS spectrum of the same material

The XRD of the particles was acquired 1 year after production and still shows a clear pattern of maghemite structure, evidence for successful maghemite stabilization (Fig. 10). The crystallite size was calculated to be 3.8 nm by use of Scherrer equation. Even though the structure is conserved, the introduction of 13% of Y by weight, of course, affects the vibrational states of the atoms (Additional file 1: Figure S3).

## Discussion

The proposed use of IONs as an iron fertilizer has been investigated before in other systems [17–19]. In this investigation, it was for the first time tested whether there is an enzymatic effect of a similar fertilizer, additional to the effect of providing the micro nutrient, iron, to an important crop species. The control was given an adequate amount of chelated iron. We also tested, a positive control, in which a molar equivalent amount of iron (III) ions was supplemented to the negative control with an adequate amount of plant available iron. Hence, the positive effects from IONs seen in our experiments arise from the properties of the IONs. We would like to suggest that it is the known enzymatic effects of IONs that are at play [23, 25, 26]. Other mechanisms can still not be excluded—IONs could also interact with proteins, lipids, and other biomolecules [27], or it can be that the nanoparticles absorb native iron ions onto the surface and hence reduce harmful Fenton reactions. The fact that the hydrogen peroxide levels of leaves were reduced in the ION-treatments is in itself an indirect proof of nanoparticle uptake. Together with increased concentration of iron and changed magnetization in maghemite the picture becomes more complete. The positive control with iron (III) ions had a reduced concentration of iron content in the leaves, indicating that the plants have capability to reduce iron ion uptake as a defense mechanism. This further suggests that the increased concentration of iron in the leaves in maghemite treatments are indeed nanoparticles, which are not as toxic to the plant as elevated levels of iron ions can be. The magnetic measurements show a superparamagnetic behavior and blocked nanoparticle magnetic moments at low temperature typical of very small γ-Fe_2_O_3_ in the leaves treated with maghemite [28], clearly demonstrating nanoparticle uptake. The miniscule amount of Y administered should not produce any effect on the plants; there is little known about the effects of Y on plants, but Fu et al. (2014) established that 2 mg L^−1^ Y was the median lethal dose (LD50) in a hydroponic system, and Maksimovic et al. (2014) started seeing toxic effects at 10^−5^ mol L^−1^ Y [29, 30]. In the highest concentration used in our experiments, an approximate amount of 50 mg (5.6 10^−4^ mol) of Y was added per pot by irrigation to the soil, from which only a fraction can be expected to be taken up. What is taken up should not be readily available as ions, but rather be bound in the maghemite particles. The purpose of introducing Y into the synthesis is to reduce the solubility of maghemite nanoparticles and also to prevent transformation into hematite, a less enzymatic form of iron oxide. Undoubtedly, it is beneficial to have increased catalase activity during stress conditions [31], since a whole range of stress conditions are known to cause toxic accumulations of H_2_O_2_ [32]_._ Further, it has become increasingly evident that H_2_O_2_ also serve as a signaling molecule for stress [32, 33]. Increased biomass production is yet to be corroborated, preferably looking into the oil seed yields and quality, as well. Other features, such as increased speed of leaf growth, a very good property in agricultural setting where competition against weeds is crucial, can readily be taken into consideration. It has been shown in *Arabidopsis* that nano zero valent iron particles can induce extrusion of protons into the apoplast of leaves and thereby allowing turgor-driven cell-wall expansion [34]. The same effect was also observed in roots, which could also be beneficial during drought stress [35]. They also observed an increase in stomatal openings of the leaves which could lead to water loss, but when they measured, there was only a marginal difference compared to the control. It is a known paradox that the relationship between stomatal opening and water transpiration is not linear [36]. This relationship is also highly affected by the environment by, for example, relative humidity or wind [37]. Although, zero valent nanoparticles are of course not to be considered the same as maghemite, the mechanism for leaf elongation seen in our experiments must be investigated. Ghafariyan et al. (2013) observed, as we did, an increase in chlorophyll concentration in the leaves upon addition of IONs compared to a negative control with no iron at all. When they compared to chelated iron there was no difference. However, adding equal amounts of chelated iron as IONs will result in more plant available iron, since in the case of particles great parts of the iron is stored in the crystal structures. Hence, there is a possibility that the plants only fertilized with IONs where actually suffering iron deficiency. We found higher amounts of chlorophyll in the leaves (according to SPAD-measurements, see Fig. 1) when IONs were added auxiliary to the chelated iron. We also measured a reduced amount of hydrogen peroxide and MDA in the leaves, after drought, when we added IONs. Rui et al. (2016) did not measure hydrogen peroxide but MDA and enzymes related to oxidative stress. They suggested that oxidative stress does not occur from addition of IONs, and indeed, they did as well find a reduced amount of MDA in the leaves, compared to chelated iron, on 10 mg kg^−1^ concentration. In the roots, they saw a reduction of MDA as they increased concentration of IONs. They also measured a reduced amount of superoxide dismutase and peroxidase activity compared to chelated iron suggesting that our hypothesis that IONs can work as reactive oxygen scavengers, in vivo, might be right. Reactive oxygen scavenging was further demonstrated by our measured reduction of hydrogen peroxide in the leaves of *Brassica napus*. This explains the increased resistance to drought that is observed upon addition of IONs.

## Conclusions

Our experiments have provided evidence for the mechanism of IONs acting as nanozymes *in planta*, revealing coupling between a decrease in hydrogen peroxide contents in the leaves of *Brassica napus* and introduction of IONs. The increased resistance to drought that is observed upon addition of IONs can thus be related to relieved oxidative stress.

## Methods

### Experimental Conditions and Design

*Brassica napus* seeds, of the spring rape variety Larissa (Scandinavian Seed AB, Lidköping, Sweden), were sterilized and germinated on agar plates for 3 days before seedlings of similar sizes were transferred to pots with sterilized S-Soil (Hasselfors garden, Örebro, Sweden). It is a soil for professional growth of seedlings with low amount of all macro and micro nutrients, perlite for aeration, growth-stimulating humic acids and a pH of 6. The plants were allowed to establish in the pots for 7 days, irrigated with deionized water. Before treatments were initiated, plants were distributed between trays, so that plant size was as consistent as possible. From day seven, after transfer to pots, the plants were irrigated with nutrient solution, nutrient solution with extra FeCl_3_ or nutrient solution with different concentrations of γ-Fe_2_O_3_ IONs. Each pot was irrigated with 40 ml every day. The plants were grown in a growth chamber with 16-h light (180 μE m^−2^ s^−1^) and 8-h darkness. Temperature was set to 25 °C during irradiation and 22° during darkness and relative humidity to 65%. Plants were grown in 8 × 8 cm pots in trays harboring eight pots each. Every treatment had two trays and 16 biological replicates. The trays were moved in a rotating order every day to compensate for any variation in the chamber. The treatment went on for 5 days adding in total 200 ml of either 0.5, 0.8, 1, or 2 mg ml^−1^, totally 100, 160, 200 or 400 mg per plant, respectively. After the 5 days of adding IONs, all treatments were watered with nutrient solution (Additional file 1: Table S1), for another 5 days before 4 days of drought was initiated. After 4 days of drought, hydrogen peroxide and lipid peroxidation measurements were performed and the plants were again watered with the same nutrient solution for 3 days to study the recovery. The experiment was replicated four times.

### Nanoparticle Synthesis and Characterization

**Fig. 11:**
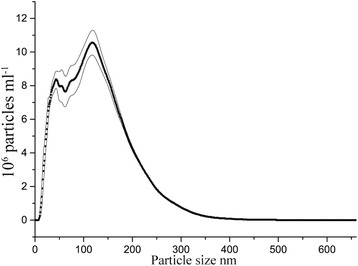
Hydrodynamic particle size distribution in water, as measured by NTA, of γ-Fe_2_O_3_ synthesized through yttrium directed sol-gel synthesis. Values are averaged from four repeated measurements, and the area within the thin lines represent mean error

**Fig. 12:**
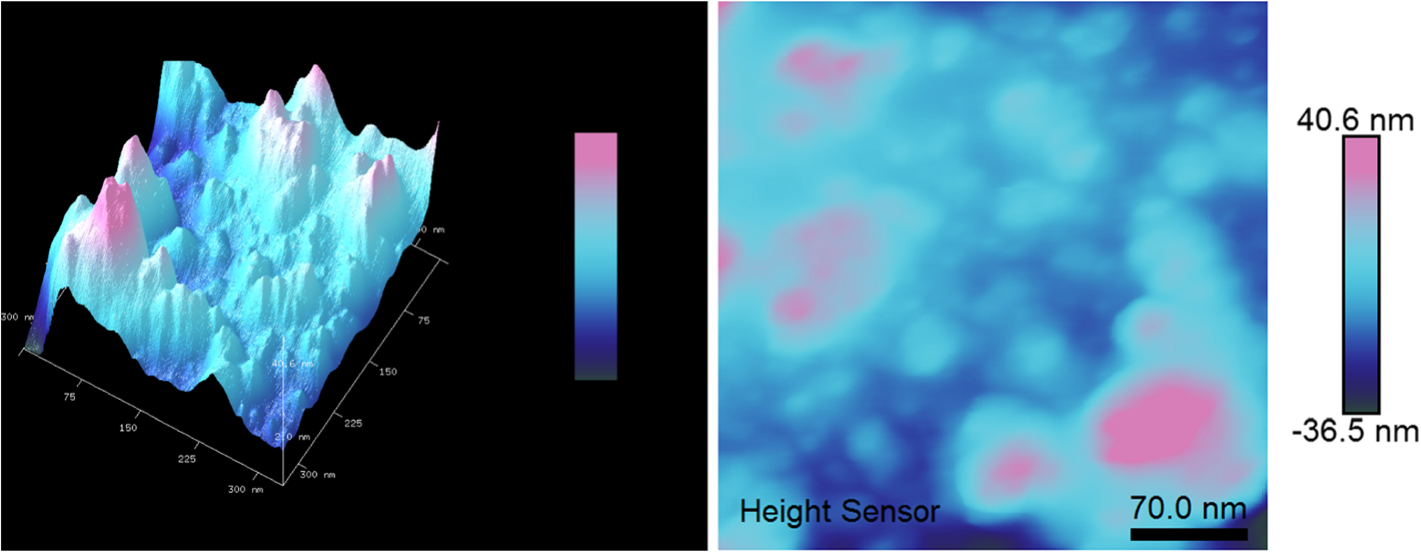
Maghemite nanoparticles synthesized through yttrium directed sol-gel, dispersed onto silicon wafer and imaged with AFM. The same image is represented in 3D and 2D

**Fig. 13:**
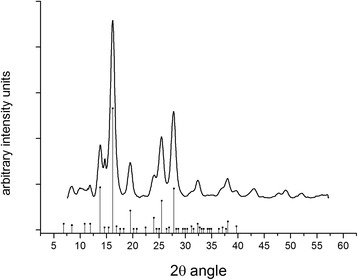
A powder diffractogram of the produced maghemite nanoparticles. The peaks align with the positions of standard maghemite from the database seen as point with drop line at the base of the figure. Crystallite size was calculated from the largest peak at 16.197 2*θ* degrees angle

The maghemite particles were produced according to the method of [38] with approximately 13% weight of yttrium (Y) and characterized by x-ray diffraction (XRD), scanning electron microscopy (SEM), nanoparticle tracking analysis (NTA), infrared spectroscopy (FTIR), thermogravimetric analysis (TGA), and atomic force microscopy (AFM). The SEM images were acquired with a Hitachi TM1000, with Oxford μDeX electron dispersive x-ray spectrometer (EDS). Hydrodynamic size was measured through nano tracking analysis (NTA) on the Nanosight 300 (Fig. 11). A Perkin-Elmer Spectrum 100 was used to do Fourier transform infrared spectroscopy (FTIR) in potassium bromide (KBr) pellets. For thermogravimetric analysis (TGA), a Perkin-Elmer Pyris 1 was used and for atomic force microscopy (AFM), a Bruker FastScan (Fig. 12). The XRD was performed on a Bruker Smart ApexII multipurpose diffractometer with molybdenum source; the crystallite size was calculated with sherrer equation using the greatest peak at 2θ° angle 16,197 with a full width half maximum (FWHM) of 1.01358489355378 calculated by Origin software peak finder function (Fig. 13). The dried IONs were suspended in a nutrient solution, with 3.4 mg L^−1^ chelated iron, the same used as control. For a complete list of all the nutrients, see Additional file 1: Table S1.

### Plant Trait Measurements

Before treatment was started, the length of the longest leaf, the first true leaf, on each plant was measured. Later, after the 5 days of sequential irrigation with IONs in nutrient solution or nutrient solution alone, the same leaf was measured again. The results are reported as percentage increase. Leaf chlorophyll was assessed by SPAD-measurements with Minolta SPAD-meter, before during and after treatment and subsequently after drought. Three measurements, on two leaves per plant, were averaged for each of the 16 biological replicates. Finally, above ground biomass of all plants were weighed up and placed into aluminum foil to be dried at 110 °C for 72 h.

### Iron Content and Magnetic Measurements

After five days of drought, the experiment was ended and all above ground plant tissue was ashed at 450 °C for 24 h. After the ash had been homogenized, 10 mg was weighed up per sample and dissolved in 3 ml of hydrochloric acid 36% on a shaker overnight. Then, the samples were diluted with 44.74 ml of 10% ethanol in Milli-Q water and subsequently measured for iron with ICP-AES at 238.204 nm. For magnetic measurements, the same ash was placed into sample holder and the exact weight for each sample was weighed up with a precision balance. Then, the sample was cooled down to 2 K, and a magnetic field sweep from 10,000 to 0 Oersted was performed on a superconducting quantum interference device (SQUID) magnetometer. The magnetic moment due to the sample holder was subtracted from the measured magnetic moment prior to normalizing with the weight of iron in the sample.

### Hydrogen Peroxide Measurements

Hydrogen peroxide in the leaves were measured by the eFOX method reported by [39]. The 16 plants were pooled into four biological replicates with four plants each. Fifty milligrams was taken from the youngest and still fresh leaf of each plant. Then, 200 mg of leaf material was powdered in a precooled mortar in liquid nitrogen. To the powder, we added 4 ml of 100 mM phosphate buffer (pH 6.9) and mortared the ice into a homogenous liquid. From this homogenous liquid, we transferred 1900 μl into a 2-ml Eppendorf tube and added 20 μl of 25 mM ferrous ammonium sulphate (Mohrs salt), 20 μl of 10 mM sorbitol, 20 μl of 10 mM xylenol orange, 20 μl of 99% ethanol, and 20 μl of 250 mM sulfuric acid. A full visible absorbance spectrum was taken for each sample, but the difference between 550 and 800 nm was used for hydrogen peroxide quantification. A calibration curve from 2 to 40 μM hydrogen peroxide was made with *R* ^2^ value of 0.9946.

### Lipid Peroxidation

Lipid peroxidation was measured according to the method of [40]. Samples were harvested in the same manner as for hydrogen peroxide measurements, except that they were homogenized in 4 ml 0.1% *w*/*v* trichloroacetic acid (TCA). The absorbance was measured at 532 nm and corrected for nonspecific turbidity by subtracting the absorbance at 600 nm. The extinction coefficient of 155 mM cm^−1^ was used to calculate malondialdehyde concentration (MDA).

### Statistical Analysis

All statistics were performed in Minitab 17 software. All data was run through a one-way ANOVA with Fisher test for grouping. Student’s *t* test was performed to find specific *p* values between groups found to have statistically significant differences.

## Additional file

Additional file 1: Supplementary material. A complete list of nutrients, in the solution, that was used as control and medium for ION application. Nitrogen is divided into ammonia and nitrate. All micronutrients are chelated. No cadmium, chloride or sodium was present. **Figure S1.** Oilseed rape plants after 5 days of drought. A. Control plants treated with nutrient solution (Table S1). B. Plants treated with nutrient solution supplemented with IONs. **Figure S2.** Two selected plots of magnetic susceptibility to demonstrate the superparamagnetic behavior present in the maghemite treatment. The selected control is of general behavior for the group while the maghemite is the sample showing most pronounced superparamagnetism. **Figure S3.** Infrared absorbance spectrum of yttrium directed maghemite nanoparticles. Wavenumber of the peaks are annotated in the graph. (DOCX 4838 kb)
