# Supplementary material for: Maghemite Nanoparticles Acts as Nanozymes, Improving Growth and Abiotic Stress Tolerance in Brassica napus
Source: Nanoscale Res Lett. 2017 Dec 19;12:631. doi: 10.1186/s11671-017-2404-2 (PMC5736512; doi:10.1186/s11671-017-2404-2)
Supplement: Supplementary file 1 — A complete list of nutrients, in the solution, that was used as control and medium for ION application. Nitrogen is divided into ammonia and nitrate. All micronutrients are chelated. No cadmium, chloride or sodium was present. Figure S1. Oilseed rape plants after 5 days of drought. A. Control plants treated with nutrient solution (Table S1). B. Plants treated with nutrient solution supplemented with IONs. Figure S2. Two selected plots of magnetic susceptibility to demonstrate the superparamagnetic behavior present in the maghemite treatment. The selected control is of general behavior for the group while the maghemite is the sample showing most pronounced superparamagnetism. Figure S3. Infrared absorbance spectrum of yttrium directed maghemite nanoparticles. Wavenumber of the peaks are annotated in the graph. (DOCX 4838 kb) [file 11671_2017_2404_MOESM1_ESM.docx]

Supplementary Material

Nanozymes, an underestimated force of nature, improving growth and abiotic stress tolerance in plants

N. G Martin Palmqvist^*^, Gulaim A. Seisenbaeva, Peter Svedlindh, Vadim G. Kessler

*** Correspondence:** Nils Gustav Martin Palmqvist, martin.palmqvist@slu.se

# Supplementary Figures and Tables

## Supplementary Figures

**Table S1.** A complete list of nutrients, in the solution, that was used as control and medium for ION application. Nitrogen is divided into ammonia and nitrate. All micronutrients are chelated. No cadmium, chloride or sodium was present.

| Nutrient | Stock conc. g/L | Final Conc. g/L |
| --- | --- | --- |
| Nitrogen | 51 | 1.02 |
| Ammonia | 20 | 0.4 |
| Nitrate | 31 | 0.62 |
| Phosphor | 10 | 0.2 |
| Potassium | 43 | 0.86 |
| Sulfur | 4 | 0.08 |
| Calcium | 3 | 0.06 |
| Magnesium | 4 | 0.08 |
| Iron | 0,17 | 0.0034 |
| Manganese | 0,2 | 0.004 |
| Boron | 0,1 | 0.002 |
| Zink | 0,03 | 0.0006 |
| Copper | 0,015 | 0.0003 |
| Molybdenum | 0,0004 | 0.000008 |


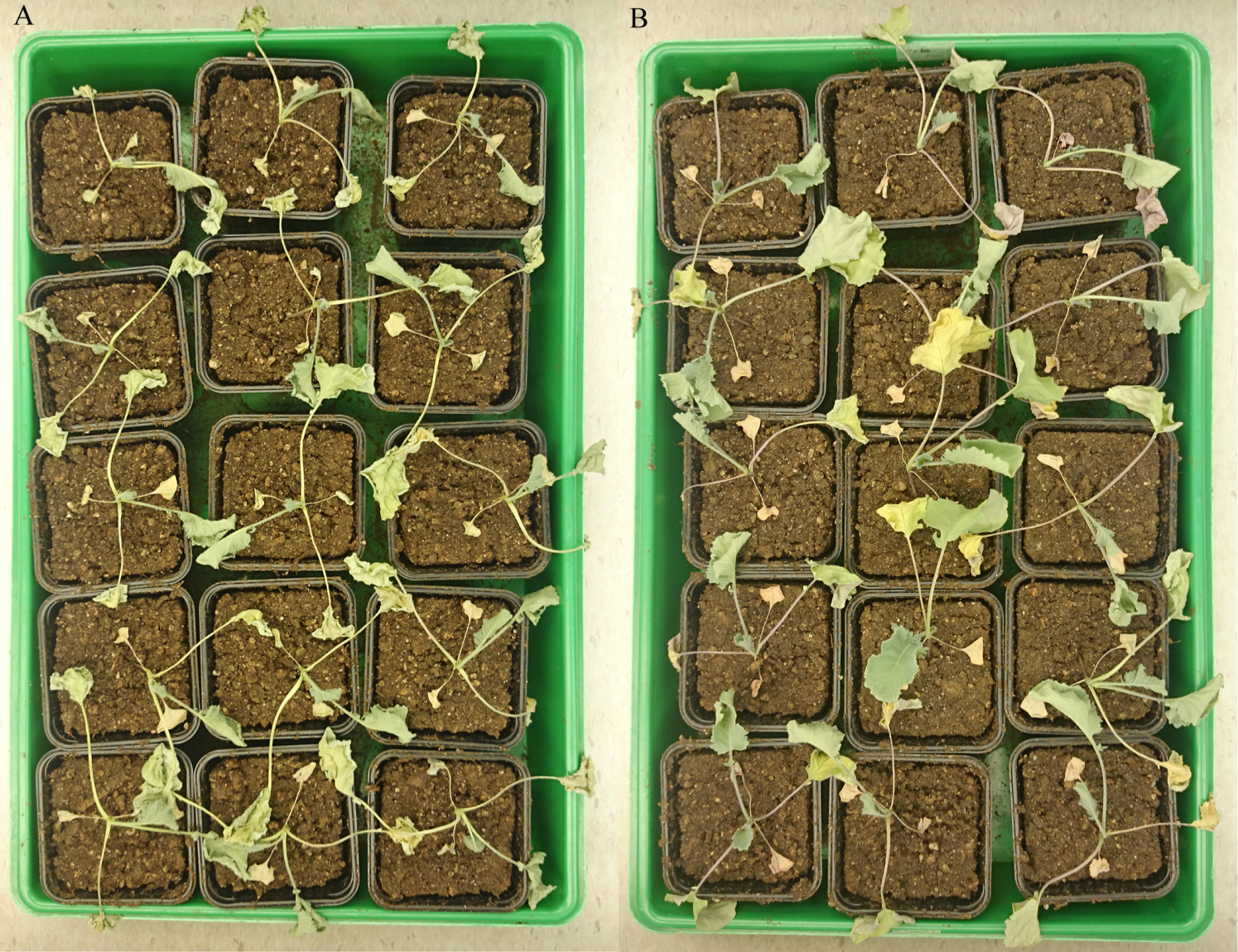


**Figure S1.** Oilseed rape plants after five days of drought. A. Control plants treated with nutrient solution (table S1). B. Plants treated with nutrient solution supplemented with IONs.


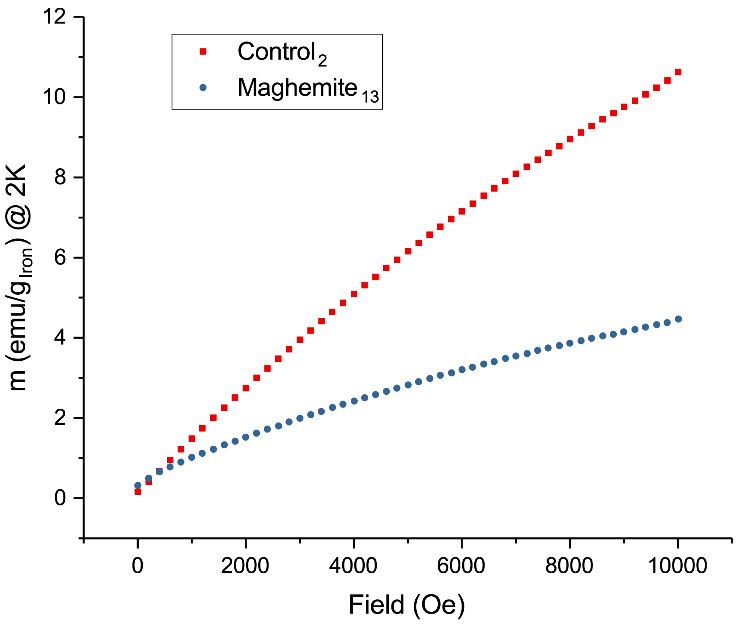


**Figure S2**. Two selected plots of magnetic susceptibility to demonstrate the superparamagnetic behavior present in the maghemite treatment. The selected control is of general behavior for the group while the maghemite is the sample showing most pronounced superparamagnetism.





**Figure S3**. Infrared absorbance spectrum of yttrium directed maghemite nanoparticles. Wavenumber of the peaks are annotated in the graph.
